# Clinical efficacy of peloidotherapy on pain, sleep quality, and central sensitization-related symptoms in chronic low back pain: a comparative analysis

**DOI:** 10.1007/s00484-026-03225-1

**Published:** 2026-05-19

**Authors:** Nihal Yılmaz, Kağan Özkuk

**Affiliations:** 1 Department of Physical Medicine and Rehabilitation, Usak University Faculty of Medicine, Atatürk Avenue, 1.Eylül Campus, UŞAK, 6400 Turkey; 2 Department of Medical Ecology and Hydroclimatology, Usak University Faculty of Medicine, Usak, Turkey

**Keywords:** Chronic Low Back Pain, Peloidotherapy, Central Sensitization, Sleep Quality, Fatigue, Disability

## Abstract

This study aimed to investigate the effect of peloidotherapy applied in chronic low back pain on pain and central sensitization and its reflections on patients’ sleep quality, fatigue levels, functional capacity, and disability status. In this prospective, randomized, single-blind study, 74 patients were assigned to the Electrotherapy Group (EG: hot pack+TENS+exercise) or Peloidotherapy Group (PG: peloidotherapy+TENS+exercise) for 15 sessions. Treatments were administered 5 days per week for 3 weeks. Clinical assessments were performed pre/post-treatment using the Visual Analog Scale (VAS), Central Sensitization Inventory (CSI), Quebec Back Pain Disability Scale (QBPDS), Functional Assessment of Chronic Illness Therapy–Fatigue (FACIT-F), Pittsburgh Sleep Quality Index (PSQI), and EQ-5D-3 L. Both groups showed significant within-group improvements in most outcomes (all *p* < 0.001), except for the EQ-5D index in the EG. According to the ANCOVA results adjusted for baseline values and pain duration, the PG demonstrated significantly superior post-treatment outcomes compared to the EG in VAS (*p* = 0.006), EQ-5D index (*p* = 0.004), and PSQI (*p* = 0.044) scores. Furthermore, the adjusted delta score analysis confirmed that the improvements in the PG were significantly greater than those in the EG for VAS (*p* = 0.026), EQ-5D (*p* = 0.004), and PSQI (*p* = 0.044). In contrast, no significant between-group differences were observed for CSI, QBPDS, FACIT, or EQ-5D VAS scores (*p* > 0.05). Both treatments improved pain and central sensitization-related measures, likely due to shared thermal effects. While peloidotherapy showed superior improvements in pain intensity, sleep quality, and health status, no specific superior effect on CSI scores was demonstrated compared to electrotherapy.

## Introduction

Low back pain (LBP) is one of the most common musculoskeletal conditions in the world and is among the leading causes of disability (Nicol et al. 2023). According to World Health Organization data, 619 million people experienced LBP in 2020, exhibiting a significant increase compared to 1990, and the number of cases is expected to increase by 2050 (Briggs et al. 2025). This trend has economic and societal impacts, which are driven by increased healthcare demand, a loss of productivity and an increasing need for rehabilitation services (Cieza et al. 2020; Ferreira et al. 2023). Although a specific cause is rarely identified, non-specific LBP remains the dominant subgroup, accounting for nearly 90% of all cases (Bardin et al. 2017). This prevalence highlights the necessity of evidence-based conservative management and precise clinical classification (e.g., exclusion of serious pathologies, assessment of radicular signs, symptom duration, etc.) (Bardin et al. 2017; Gianola et al. 2022; Ferreira et al. 2023). While the prognosis is generally favorable, LBP can transition into a chronic state if not addressed with necessary treatment (Menezes Costa et al. 2012; Nicol et al. 2023).

According to the ACTTION-APS Pain Taxonomy (AAPT), chronic low back pain (CLBP) is defined as pain localized between the inferior margin of the 12th ribs and the inferior gluteal folds, with or without referral to the proximal lower extremities (Markman et al. 2020). To meet the diagnostic criteria for chronicity, the pain must be present on most days for at least three months, or for at least half of the days during the preceding six months. According to this definition, CLBP is no longer viewed merely as a symptom of an underlying lesion but as a complex, multifactorial, and persistent condition maintained by interacting neurobiological, psychological, and social processes (Markman et al. 2020; Li et al. 2021; Nicol et al. 2023; Zhou et al. 2024). The pathophysiology of CLBP involves prolonged inflammatory and neurogenic signaling that facilitates peripheral sensitization and central sensitization (CS), alongside impaired descending inhibitory modulation, consequently lowering nociceptor thresholds and increasing spinal and supraspinal neuronal excitability (Nijs et al. 2010; Bushnell et al. 2013; Li et al. 2021). These processes can lead to long lasting sensitization of nociceptors, reduced neuronal excitation thresholds, and consequently the emergence of different clinical manifestations (Nijs et al. 2010; Bushnell et al. 2013; Li et al. 2021). Peripheral sensitization is driven by sustained inflammatory mediator activity that lowers nociceptor excitation thresholds, producing hypersensitivity to otherwise innocuous stimuli (Nijs et al. 2010). CS is characterized by increased neuronal excitability at spinal and supraspinal levels, synaptic plasticity changes, and reduced inhibitory control; mechanistic descriptions commonly include NMDA receptor dependent facilitation, receptive field expansion, and temporal summation (wind up) (Nijs et al. 2010; Li et al. 2021). As a result, hyperalgesia, allodynia, and spontaneous pain may occur together with dysfunction in descending inhibitory pathways that modulate pain. Beyond amplifying pain, central mechanisms are also linked to disruption of cognitive and emotional pain control and to adverse outcomes in sleep quality, fatigue, mood, and cognitive functioning through altered corticolimbic processing (Bushnell et al. 2013; Finan et al. 2013). Clinically, persistence of these processes is associated with marked reductions in functional capacity and increases in disability. In this context, the Central Sensitization Inventory (CSI) is frequently used to screen central sensitivity symptom burden; a commonly used threshold is CSI ≥ 40 (Neblett et al. 2013), and among LBP populations the proportion meeting this threshold has been reported to range widely from 18.3% to 78.2%, underscoring substantial clinical heterogeneity (Opara et al. 2025).

Due to the CLBP’s significant contribution to the global burden of disability, effective rehabilitation strategies are critical for decreasing its individual and societal impacts. Therefore, effective management requires evidence-based conservative care and a structured clinical approach. This process guides classification and treatment through the assessment of symptom duration, suspected underlying causes, the presence of radicular symptoms, and relevant anatomical or radiographic findings (Qaseem et al. 2017). The main objectives of treatment are to reduce pain, increase physical activity and function, and minimize disability (Qaseem et al. 2017). There are both pharmacological and non-pharmacological options available for treating CLBP. These include medical treatment, exercise therapy, physiotherapy, manual therapy, acupuncture, balneotherapy and peloidotherapy. Balneological interventions are gaining prominence as conservative treatment modalities. Their therapeutic potential is attributed to thermotherapeutic and anti-inflammatory effects, alongside modulation of stress and immune responses (Fioravanti et al. 2011; Gálvez et al. 2018). Current evidence from systematic reviews suggests that balneotherapy may improve pain intensity and functional limitations in individuals with CLBP (Bai et al. 2019; Forestier et al. 2022; Mao et al. 2023; Crevenna et al. 2025).

Clinical studies in CLBP indicate that peloidotherapy may be associated with improvements in pain, function, and quality of life, yet the underlying mechanisms are not fully understood. Evidence remains limited regarding whether peloidotherapy modifies CS and how such effects relate to patient centered outcomes including sleep quality, fatigue, functional capacity, and disability (Yücesoy et al. 2019, 2021; Hahm et al. 2020; Karaarslan et al. 2021).

Accordingly, in this study we aimed to investigate the effect of peloidotherapy applied in CLBP on pain and CS and to examine its reflections on patients’ sleep quality, fatigue levels, functional capacity, and disability status.

## Material and method

### Study design

This prospective randomized controlled trial was conducted in the Department of Physical Medicine and Rehabilitation and the Department of Medical Ecology and Hydroclimatology at Uşak Training and Research Hospital, following approval from the Ethics Committee.

### Study setting and participants

Between December 2024 and April 2025, 74 patients diagnosed with non-specific CLBP who met the study criteria were included. All patients were evaluated by the investigating physician prior to the treatment.

The inclusion criteria were as follows: (1) patients aged 40–75 years; (2) a pain Visual Analogue Scale (VAS) score during activity > 40 mm at the baseline examination; and (3) a diagnosis of CLBP as defined by the AAPT criteria (Markman et al. 2020).

Exclusion criteria were as follows: chronic lumbosacral radicular pain (e.g., radiculopathy or sciatica); neurogenic claudication associated with lumbar spinal stenosis; spondylolisthesis; pain due to spinal osteoporosis or acute vertebral fracture; inflammatory LBP (e.g., axial spondyloarthropathy); a history of lumbar surgery; inflammatory rheumatic diseases; psychiatric or neurological disorders; uncontrolled arterial hypertension; decompensated organ failure; malignancy; infectious diseases; and pregnancy. Additionally, patients were excluded if they had received systemic steroid treatment within the previous three months; experienced major trauma or undergone surgery within the past six months; or received physical therapy, peloidotherapy, or balneotherapy within the past six months.

### Randomization and blinding

74 patients who met the study criteria and signed the informed consent form prior to the study were included. Using a random number table generated by a computer, patients were divided into two groups by simple randomization. Due to the nature of the treatment, patient blinding was not possible. However, the outcome assessment process was blinded. The evaluation of patients and the statistical analysis of the results were performed by a physician and a biostatistics specialist who were unaware of the patients’ treatments and group allocations.

### Interventions

All patients received treatment for 3 weeks (5 days per week, a total of 15 sessions). All procedures were performed under physician supervision and with the assistance of experienced physiotherapists.

In the Electrotherapy Group (EG), each patient received 20 min of local hot pack application at 45 °C and transcutaneous electrical nerve stimulation (TENS) at 80 Hz, followed by 15 min of range of motion, stretching, and isometric exercises (hamstring, pelvic, and abdominal muscles) in each session.

In the Peloidotherapy Group (PG), patients received 20 min of peloidotherapy, followed by 20 min of conventional TENS at 80 Hz and 15 min of range of motion, stretching, and isometric exercises (hamstring, pelvic, and abdominal muscles).

For patients in the PG, peloid containing Pelomin^®^, obtained from the Tuzla thermal springs, was applied to the lumbar region (lower border: iliac crest, upper border: costal arch, lateral borders: posterior superior iliac spines) at a temperature of 44–45 °C and a thickness of approximately 2 cm. The application area was then covered with a nylon sheet and wrapped with a towel. After 20 min, the peloid was removed using soft cloths moistened with warm water.

The peloid was prepared by mixing sepiolite clay with Tuzla Spa mineral water, which contains high concentrations of chloride, sulfate, magnesium, and calcium, with a total mineralization of 4,145 mg/L. The total mineralization of the peloid was 3,406 mg/L (Özkuk et al. 2017).

The use of non-steroidal anti-inflammatory drugs and muscle relaxants was prohibited throughout the treatment period. Participants were clearly informed of this restriction at the time of enrollment. Only paracetamol was permitted as rescue analgesia when necessary, with a maximum daily dose of 2 g.

### Outcome measures

All participants were evaluated before and after treatment using the pain Visual Analog Scale (VAS), EuroQol-5D-3 L (EQ-5D-3 L), EQ-Visual Analog Scale (EQ-VAS), CSI, Quebec Back Pain Disability Scale (QBPDS), Functional Assessment of Chronic Illness Therapy (FACIT) -Fatigue Index, and Pittsburgh Sleep Quality Index (PSQI).

VAS is a horizontal 100-mm straight line used to determine the severity of pain. A score of “0” indicates no pain, while “100” indicates very severe pain. Patients were asked to evaluate the severity of their pain at rest, during activity, and at night (McCormack et al. 1988).

The EQ-5D is a self-report scale developed by the EuroQol Group, a Western European research consortium on quality of life. The five dimensions of mobility, self-care, usual daily activities, pain/discomfort, and anxiety/depression—are each assessed with one question. Each dimension has three response options. These five dimensions generate an index score ranging from − 0.59 to 1. In addition, the scale includes a VAS (EQ-VAS), scored between 0 and 100, where 0 represents “the worst imaginable health state” and 100 represents “the best imaginable health state” (Süt and Ünsar 2011).

The QBPDS evaluates the extent to which LBP affects daily life activities. Its Turkish validity and reliability were established (Melikoglu et al. 2009). In this scale, the difficulty caused by back pain is assessed across 20 different activities. The scoring ranges from 0 to 5, where “0” indicates “not difficult at all” and “5” indicates “unable to do”.

The CSI: Part A of the CSI includes all symptoms of CS syndrome (CSS) and serves as a tool to assist clinicians in identifying patients with CSS. A score above 40 is considered indicative of CS (Keleş et al. 2021).

The FACIT Fatigue Index is a 13-item patient-reported outcome measure assessing fatigue over the previous seven days. Each item is rated on a 5-point Likert scale ranging from 0 (not at all) to 4 (very much). Some items are reverse scored, and a total score is calculated by summing all items, yielding a score range of 0–52, with higher scores indicating less fatigue (Çinar and Yava 2018).

The PSQI is a 19-item self-report scale that evaluates sleep quality and disturbances during the past month. It contains 24 questions in total, of which 19 are self-report items and 5 are to be answered by a bed partner or roommate. Each component is rated on a 0–3 scale. The total score ranges from 0 to 21, with scores greater than 5 indicating “poor sleep quality” (Ağargün et al. 1996).

The primary outcome of the study was pain intensity measured using the VAS, which was also used for the sample size calculation. Secondary outcomes included CS and other clinical parameters related to sleep quality, fatigue, functional capacity, disability, and quality of life.

### Sample Size

Sample size calculation was performed using the VAS scores from the study conducted by Hahm et al. in patients with CLBP. The effect size was determined to be 0.8363145. With an α error probability of 0.05 and a power of 0.95, it was calculated that at least 32 patients were required in each group (Hahm et al. 2020).

### Statistical analysis

Continuous variables were summarized as mean ± standard deviation and/or median (min–max), whereas categorical variables were presented as counts and percentages. Normality was assessed using the Kolmogorov–Smirnov goodness-of-fit test. Between-group comparisons were performed using Student’s t-test for normally distributed variables and the Mann–Whitney U test for non-normally distributed variables. Within-group comparisons were conducted using the paired-samples t-test. Categorical variables were compared using the chi-square test or Fisher’s exact test, as appropriate. Analysis of Covariance (ANCOVA) was used for between-group comparisons of post-treatment scores and delta (change) scores (after confirming that parametric assumptions were met), with baseline scores and symptom duration (pain duration) included as covariates to adjust for pre-existing differences between the groups. The adjusted results were presented as estimated marginal means with standard errors and 95% confidence intervals. All analyses were performed using IBM SPSS Statistics, version 26.0 (IBM Corporation, Armonk, NY, USA). A p-value < 0.05 was considered statistically significant.

## Results

**Fig. 1:**
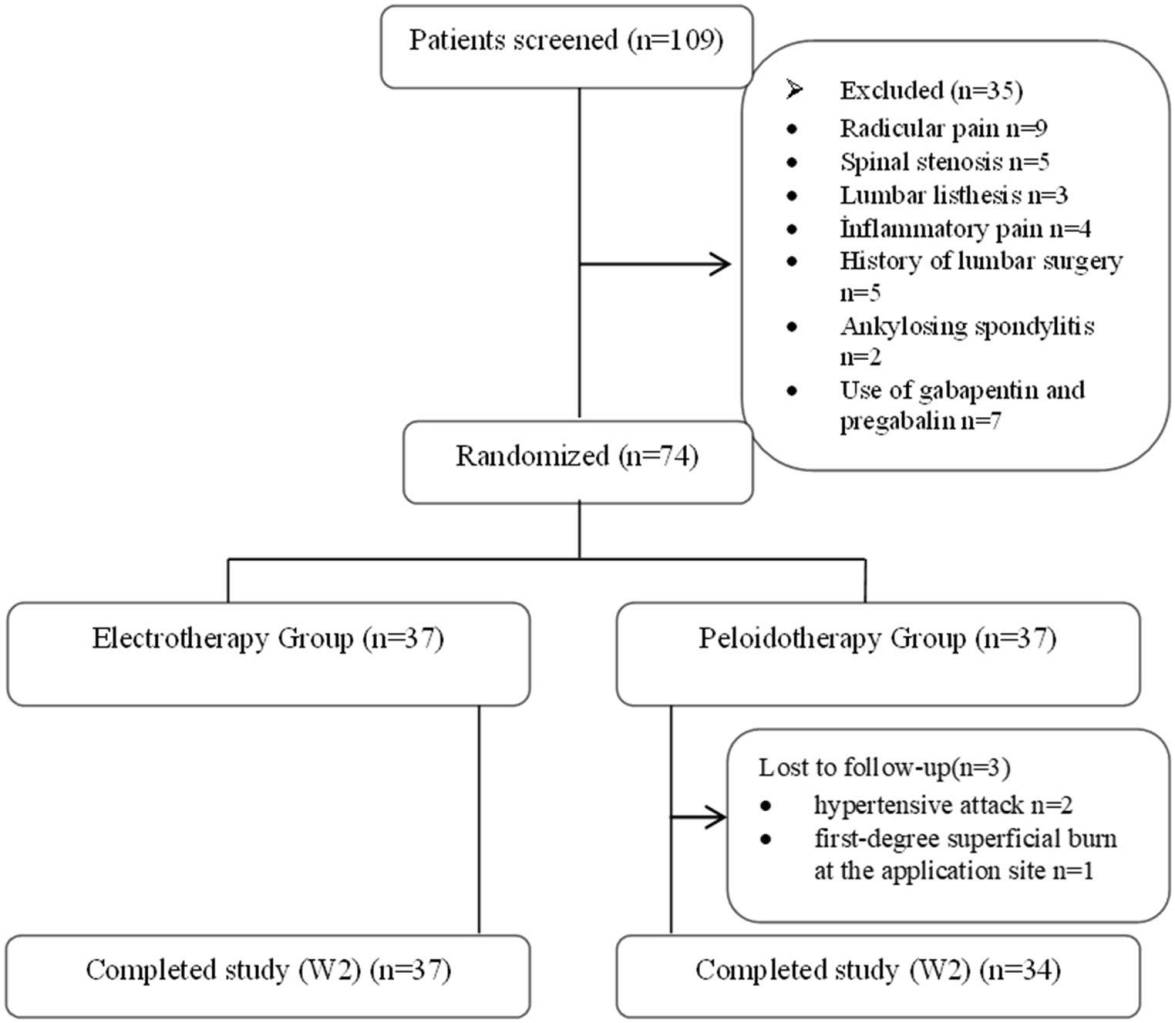
Flow diagram of participants in the study

Between December 1, 2024, and April 30, 2025, 109 patients diagnosed with CLBP were evaluated for eligibility. Of these, 35 patients were excluded due to not meeting the inclusion criteria (radicular pain *n* = 9, spinal stenosis *n* = 5, spondylolisthesis *n* = 3, inflammatory pain *n* = 4, lumbar surgery *n* = 5, ankylosing spondylitis *n* = 2, use of gabapentin and pregabalin *n* = 7). Consequently, 74 patients who met the inclusion criteria were accepted. During the treatment period, 3 participants from the PG (hypertensive crisis *n* = 2, first-degree superficial burn at the application site *n* = 1) withdrew from the study. Although transient adverse events leading to withdrawal occurred in three patients in the PG, no permanent or life-threatening complications were observed. The study flow diagram is presented in Fig. 1

There were no statistically significant differences found between the groups regarding demographic characteristics. However, pain duration was significantly longer in the EG compared to the other group (*p* = 0.036) (Table 1).

**Table 1:** Comparison of the groups according to age, sex, disease duration, and presence of chronic disease

	Electrotherapy Group (*n* = 37)	Peloidotherapy Group (*n* = 34)	*p*-value
Age (years) (Means ± SD)	63.43±12.0	62.50±7.7	0.702*
Duration (months) [median (min–max)]	36 (6–180)	12 (12–360)	0.036**
Sex			0.084***
– Female	26 (70.3%)	30 (88.2%)	
– Male	11 (29.7%)	4 (11.8%)	
Diabetes Mellitus	17 (45.9%)	16 (47.1%)	0.925****
Hypertension	13 (35.1%)	9 (26.5%)	0.430****
Coronary artery disease	6 (16.2%)	3 (8.8%)	0.482***
Pulmonary disease	4 (10.8%)	1 (2.9%)	0.359***
Chronic renal failure	2 (5.4%)	1 (2.9%)	1.000***
Hypothyroidism	0 (0.0%)	2 (5.9%)	0.226***
Anemia	2 (5.4%)	3 (8.8%)	0.665***
Liver disease	2 (5.4%)	3 (8.8%)	0.665***
Gastric disease	1 (2.7%)	0 (0.0%)	1.000***
Epilepsy	0 (0.0%)	2 (5.9%)	0.226***

*Student’s t-test

**Mann-Whitney U test

***Fisher’s Exact Test

****Chi-square test

Within-group analyses showed that both groups demonstrated significant within-group improvements in most outcomes. In the EG, VAS, CSI, QBPDS, and FACIT scores decreased significantly after treatment (all *p* < 0.001), and PSQI scores also improved (*p* = 0.001). EQ-5D VAS increased significantly (*p* < 0.001), whereas the change in the EQ-5D index was not significant (*p* = 0.173). In the PG, post-treatment scores improved across all assessed domains: VAS, CSI, QBPDS, FACIT, and PSQI decreased (all *p* < 0.001), while both EQ-5D index and EQ-5D VAS increased (both *p* < 0.001) (Table 2).

**Table 2:** Within-group changes and between-group comparisons of scale scores adjusted for baseline values and pain duration

Outcome	Group	Pre-treatment (Mean ± SD)	Post-treatment (Mean ± SD)	*p* (Within-group)^a^	Adjusted Post-treatment (Mean ± SE) ^b^	95% CI	F value	*p* (Between-group)^c^
VAS	EG	7.29 ± 1.77	4.72 ± 1.50	< 0.001	4.74 ± 0.30	4.13 to 5.36	7.898	**0.006**
	PG	7.38 ± 1.59	3.50 ± 2.23	< 0.001	3.48 ± 0.32	2.85 to 4.13		
EQ-5D	EG	0.35 ± 0.25	0.43 ± 0.23	0.173	0.43 ± 0.03	0.37 to 0.49	8.924	**0.004**
	PG	0.34 ± 0.19	0.56 ± 0.13	< 0.001	0.57 ± 0.33	0.50 to 0.63		
EQ-5D VAS	EG	55.94 ± 18.02	73.78 ± 15.60	< 0.001	72.46 ± 2.40	67.68 to 77.25	1.661	0.202
	PG	46.17 ± 20.70	75.58 ± 14.49	< 0.001	77.02 ± 2.50	72.03 to 82.03		
CSI	EG	46.86 ± 18.32	35.48 ± 19.31	< 0.001	34.66 ± 2.43	29.79 to 39.52	0.629	0.431
	PG	43.76 ± 12.79	30.94 ± 13.82	< 0.001	31.86 ± 2.54	26.77 to 36.92		
QBPDS	EG	45.91 ± 17.65	37.21 ± 19.44	< 0.001	37.71 ± 2.35	33.02 to 42.40	0.717	0.400
	PG	48.47 ± 17.57	35.35 ± 18.31	< 0.001	34.82 ± 2.45	29.93 to 39.71		
FACIT	EG	26.81 ± 10.56	20.00 ± 9.58	< 0.001	19.77 ± 1.22	17.33 to 22.21	0.980	0.326
	PG	25.94 ± 7.58	17.76 ± 8.35	< 0.001	18.01 ± 1.28	15.47 to 20.56		
PSQI	EG	9.94 ± 4.11	7.70 ± 4.33	0.001	7.18 ± 0.55	6.09 to 8.27	4.213	**0.044**
	PG	7.79 ± 3.37	4.94 ± 3.05	< 0.001	5.51 ± 0.57	4.38 to 6.65		

*SD* Standard Deviation, *SE *Standard Error, *CI *Confidence Interval, *EG *Electrotherapy Group, *PG *Peloidotherapy Group, *VAS *Visual Analog Scale, *CSI *Central Sensitization Inventory, *QBPDS *Quebec Back Pain Disability Scale, *FACIT *Functional Assessment of Chronic Illness Therapy, *PSQI *Pittsburgh Sleep Quality Index, *EQ-5D *EuroQol-5. ^a^ Paired samples t-test was used for within-group comparisons (pre-treatment vs. post-treatment). ^b^ Estimated Marginal Means (Adjusted Mean) represent the predicted post-treatment values when baseline scores and “Pain Duration” are controlled as covariates. ^c^ ANCOVA (Analysis of Covariance) was performed for between-group comparisons of post-treatment scores, with baseline scores and pain duration included as covariates to adjust for pre-existing differences

Between-group comparisons adjusted for baseline values and pain duration demonstrated that post-treatment VAS scores were significantly lower in the PG than in the EG (F = 7.898, *p* = 0.006). Similarly, post-treatment EQ-5D index scores were significantly higher in the PG compared with the EG (F = 8.924, *p* = 0.004). No significant between-group differences were observed for EQ-5D VAS, CSI, QBPDS, or FACIT after adjustment (all *p* > 0.05). However, post-treatment PSQI scores remained significantly lower in the PG than in the EG (F = 4.213, *p* = 0.044) (Table 2).

Change-score analyses adjusted for baseline values and pain duration also revealed a greater reduction in VAS scores in the PG compared with the EG (F = 5.206, *p* = 0.026). In addition, the increase in EQ-5D index scores was significantly greater in the PG (F = 8.924, *p* = 0.004). No significant between-group differences were detected for adjusted delta values of EQ-5D VAS, CSI, QBPDS, or FACIT (all *p* > 0.05). However, the reduction in PSQI scores was significantly greater in the PG than in the EG (F = 4.213, *p* = 0.044) (Table 3).

**Table 3:** Comparison of raw and adjusted delta scores of the scales between groups

Outcome	Group	Raw-Delta (Mean ± SD)	Adjusted-Delta (Mean ± SE)	95% CI	F value	*p*
VAS	EG	−2.56 ± 1.32	−2,61 ± 0.37	−3.35 to −1.87	5.206	0.026
	PG	−3.88 ± 2.91	−3,89 ± 0.39	−4.61 to −3.07		
EQ-5D	EG	0.08 ± 0.36	0.08 ± 0.03	0.02 to 0.14	8.924	0.004
	PG	0.21 ± 0.21	0.21 ± 0.03	0.15 to 0.28		
EQ-5D VAS	EG	17.83 ± 15.29	21.20 ± 2.40	16.41 to 25.98	1.661	0.202
	PG	29.41 ± 23.79	25.26 ± 2.51	20.76 to 30.76		
CSI	EG	−11.37 ± 15.32	−10.72 ± 2.44	−15.59 to −5.86	0.629	0.431
	PG	−12.82 ± 17.16	−13.54 ± 2.54	−18.61 to −8.46		
QBPDS	EG	−8.70 ± 13.50	−9.43 ± 2.35	−14.12 to −4.75	0.717	0.400
	PG	−13.11 ± 16.93	−12.32 ± 2.45	−17.21 to −7.43		
FACIT	EG	−6.81 ± 6.94	−6.62 ± 1.22	−9.06 to −4.18	0.980	0.326
	PG	−8.17 ± 9.55	−8.38 ± 1.28	−10.93 to −5.83		
PSQI	EG	−2.24 ± 3.58	−1.74 ± 0.55	−2.83 to −0.65	4.213	0.044
	PG	−2.85 ± 3.68	−3.40 ± 0.57	−4.54 to −2.26		

*SD *Standard Deviation, *SE *Standard Error, *CI *Confidence Interval, *EG *Electrotherapy Group, *PG *Peloidotherapy Group. *VAS *Visual Analog Scale, *CSI *Central Sensitization Inventory, *QBPDS *Quebec Back Pain Disability Scale, *FACIT *Functional Assessment of Chronic Illness Therapy, *PSQI *Pittsburgh Sleep Quality Index, *EQ-5D *EuroQol-5. Raw-Delta: The unadjusted change score (Post-treatment minus Pre-treatment). Adjusted-Delta: The estimated marginal means of the change scores derived from the ANCOVA model. ANCOVA: Between-group comparisons were performed with baseline scores and pain duration included as covariates to adjust for pre-existing differences. p: Represents the statistical significance between groups after adjusting for the specified covariates

## Discussion

In this study investigating the effect of hot pack + TENS + exercise therapy versus peloidotherapy + TENS + exercise therapy on pain and CS in patients with CLBP, and the reflections of this effect on sleep quality, fatigue level, functional capacity, and disability status, overall improvements were observed in both groups. Within-group evaluations, significant improvements were found in all assessment parameters in the EG, except for EQ-5D, compared to pre-treatment. When post-treatment scores were examined, VAS, EQ-5D, and PSQI values were statistically significantly better in the PG compared to the EG. After adjustment for baseline values and pain duration using ANCOVA, post-treatment VAS, EQ-5D index, and PSQI scores remained significantly better in the PG compared with the EG, whereas no significant between-group differences were observed for EQ-5D VAS, CSI, QBPDS, or FACIT. According to the delta scores between groups, statistically significant improvements were observed in VAS and EQ-5D index scores in the PG compared with the EG after adjustment, whereas no statistically significant differences were detected for EQ-5D VAS or the other parameters. Although improvements were observed across most outcome measures, changes in CSI, QBPDS, and FACIT did not differ significantly between groups. These findings suggest that replacing hot pack application with peloidotherapy may offer superior benefits in pain relief, sleep quality, and general quality of life index, whereas both thermal modalities appear similarly effective when combined with TENS and exercise in mitigating CS, functional disability, and fatigue in patients with CLBP.

Conventional physical therapy modalities used in the treatment of CLBP, including hot pack, TENS, and exercise applications, have positive effects on pain control, tissue relaxation, increased microcirculation, and central pain modulation when used alone or in combination. Hot pack application, as a superficial heat agent, leads to local vasodilation; thus, by increasing blood flow and metabolic activity, it reduces pain and muscle spasm (Nadler et al. 2004; Kominami et al. 2022). TENS provides pain modulation through mechanisms of gate control theory and endogenous opioid release (Vance et al. 2014). It is reported that TENS is an effective method for reducing pain in patients with LBP, either alone or in combination with other therapies (Karisa et al. 2023; Wahyono et al. 2024). The addition of exercise therapy may contribute to breaking the pain-spasm-pain cycle and promoting functional improvement by increasing muscle strength, enhancing flexibility, and enriching proprioceptive inputs (Hayden et al. 2021; Fernández-Rodríguez et al. 2022). In this study, the combination of hot pack, TENS, and exercise applied in the EG provided significant improvements in all parameters except the EQ-5D. This finding is consistent with existing literature data indicating that a multimodal treatment approach is more effective than a single treatment modality in CLBP (Kamper et al. 2015).

In the PG receiving TENS, exercise, and peloidotherapy, significant improvements were observed particularly in pain severity, quality of life, and sleep quality. These findings support other studies suggesting that peloidotherapy may be an effective treatment method for improving function, perception of pain, and quality of life in the treatment of CLBP (Karagülle and Karagülle 2015; Yücesoy et al. 2019, 2021; Bai et al. 2019; Hahm et al. 2020; Karaarslan et al. 2021; Forestier et al. 2022; Crevenna et al. 2025). The mechanism of action of peloidotherapy can be explained by the thermal and chemical properties of its organic and inorganic components. It has been reported that peloids exert a long-lasting thermal effect at the site of application, inducing vasodilation in deep tissues, accelerating local metabolism, and exerting an anti-inflammatory effect by decreasing proinflammatory cytokines (IL-1, TNF-α) and increasing antiinflammatory cytokine levels, thereby modulating the inflammatory response (Fioravanti et al. 2011; Ortega et al. 2017; Yücesoy et al. 2021). In peloidotherapy, the reduction of inflammation has been suggested as one of the treatment mechanisms that alleviate pain and improve functionality (Ortega et al. 2017). The thermal effect of peloidotherapy has been reported to activate TRPV1 receptors, contributing to nociceptive signal transmission, as these receptors are localized in primary afferent neurons and the central nervous system (Malanga et al. 2015; Wang et al. 2021; Zanoli et al. 2024). However, controlled and prolonged heat application may lead to desensitization of TRPV1, thereby reducing hyperalgesia and allodynia, which play a role in the process of CS. In this study, significant within-group improvements were observed in both groups. However, no significant between-group difference was detected in CSI, QBPDS, and FACIT scores. This suggests that the observed changes in CS related outcomes, functional disability, and fatigue levels may be related to non-specific effects of multimodal treatment. In particular, the thermal component of peloidotherapy, together with the heat application in the other group, may have produced comparable physiological effects, which could have limited the detection of between-group differences. Additionally, the relatively short follow-up period may have restricted the observation of more distinct and sustained neurophysiological and functional changes.

CS observed in CLBP has been reported to negatively affect quality of life by leading to increased perception of pain, sleep disturbances, and fatigue due to excessive central processing of pain signals (Nijs et al. 2010). The bidirectional relationship between sleep disturbance and chronic pain may provide an important perspective for understanding the mechanism of action of peloidotherapy (Finan et al. 2013). The anti-inflammatory and neuromodulatory properties of peloidotherapy may contribute to overall reductions in pain perception and associated functional limitations. This analgesic effect may contribute to improvements in sleep quality through the interaction between pain reduction and sleep regulation systems. Significant improvements observed in VAS and PSQI scores may indicate that the intervention contributed to improvements in both pain perception and sleep quality. Nevertheless, both groups showed comparable improvements in CSI, disability, and fatigue scores under the study conditions, without a significant between-group difference. These findings are consistent with the hypotheses proposed by Finan et al., suggesting that sleep disturbance may impair endogenous pain inhibitory mechanisms and that interventions targeting sleep disturbance in chronic pain management may enhance therapeutic efficacy (Finan et al. 2013). Therefore, the improvements observed in pain severity and sleep quality in this study should be interpreted primarily as clinical treatment effects of a combined therapeutic approach, reflecting the comparable impact of both peloidotherapy and hot pack application on CS-related outcomes.

This study has several limitations. The lack of long-term follow-up after treatment prevented us from evaluating whether the observed improvements were sustained over time. Furthermore, the relatively small sample size may have limited our ability to detect more subtle between-group differences. Finally, due to the nature of the interventions, double blinding was not feasible. In addition, the absence of a thermal-matched active control group limits our ability to isolate the specific effects of the intervention beyond thermal input.

## Conclusion

In conclusion, adding peloidotherapy to TENS and exercise may provide additional benefits in pain reduction and perceived health status in patients with CLBP. These effects may be mediated through the thermophysical and biochemical properties of peloids, potentially supporting pain–spasm-pain cycle disruption by attenuating proalgesic inflammatory activity, alleviating paravertebral muscle spasm, and influencing pain modulation pathways related to CS. Further studies with larger samples and long-term follow-up are warranted to clarify the durability of effects and the underlying mechanisms.

## Data Availability

The data supporting the findings of this study are available from the corresponding author, [KÖ], upon reasonable request.
